# The Revival of Bad Methods

**Published:** 1898-04

**Authors:** 


### THE REVIVAL OF BAD METHODS.

Attention is specially called to the article by Professor C. N. Peirce, entitled "The Past and Present in Dentistry," and particularly to that portion giving facts in relation to the action of a certain board of examiners in passing, with effusive compliments, a student in attendance at a college in the freshman year.

This board has been very pronounced in its efforts to raise (?) the standard of dental education, so much so that it has proposed to turn down all colleges that decline to come up to its standard. The facts as stated by Professor Peirce will enable the dental profession to come to correct conclusions.

If this were the only board guilty of this it might be passed over, but it is well known that it is not exceptional, though, possibly, more offensive than some others.

The National Association of Dental Faculties in 1884 did away, it was supposed forever, with the right to graduate on five years' practice. That it has come to be revived by a board of examiners will be a positive shock to the dental educators of the country, and should open their eyes to future possibilities of evil, in connection with college work, through the maladministration of law.

